# Associations of anthropometric adiposity indexes with hypertension risk: A systematic review and meta-analysis including PURE-China

**DOI:** 10.1097/MD.0000000000013262

**Published:** 2018-11-30

**Authors:** Guijuan Deng, Lu Yin, Weida Liu, Xiaoyun Liu, Quanyong Xiang, Zhenzhen Qian, Juntao Ma, Hui Chen, Yang Wang, Bo Hu, Wei Li, Yu Jiang

**Affiliations:** a State Key Laboratory of Cardiovascular Disease, Fuwai Hospital, National Center for Cardiovascular Diseases; b School of Public Health, Peking Union Medical College & Chinese Academy of Medical Sciences, Beijing; c Jiangsu Province Center for Disease Control & Prevention; d Jiangxinzhou Community Health Service Center, Nanjing; e Academy of Military Sciences, People's Liberation Army of China, Beijing, China.

**Keywords:** body mass index, hypertension, meta-analysis, systematic review, waist circumstance, waist-to-height ratio, waist-to-hip ratio

## Abstract

**Background and objective:**

The association between hypertension and obesity has been confirmed, while no agreement has been reached about which anthropometric adiposity index is the best. This meta-analysis aimed to perform a systematic review and meta-analysis on the associations of hypertension risk with body mass index (BMI), waist circumstance (WC), waist-to-hip ratio (WHR), and waist-to-height ratio (WHtR), and a prospective urban and rural epidemiology study from China (PURE-China) was added into this meta-analysis as an individual study.

**Methods:**

Systematic literature searching was conducted to identify relevant articles published up to September 2018 in CNKI, WANFANG Data, Web of Science, SinoMed, PubMed, MEDLINE, EMBASE, Cochrane Library and cross-referencing. Literature reporting the association of hypertension risk with BMI, WC, WHR, and WHtR were defined as eligible. PURE-China data were analyzed and included as 1 eligible study into meta-analyses. Summary odds ratio (OR) and area under receiver operating characteristic curve (AUC) were pooled using meta-analysis methods. Heterogeneity and publication bias were evaluated. Subgroups based on gender, country and study design were conducted as well.

**Results:**

Thirty-eight original articles including PURE-China were included into meta-analyses, involving 309,585 subjects. WHtR had the strongest association with hypertension risk (OR, 1.68; 95% confidence interval, [CI]:1.29–2.19) and prediction ability (AUC, 70.9%; 95% CI: 67.8%–74.2%), which were also confirmed in subgroup analyses based on gender and country. However, BMI was found to have the highest prediction ability in adjusted models of PURE-China and followed WC, both of which were superior to WHtR (73.7% and 73.4% vs 73.2%).

**Conclusions:**

Our overall meta-analysis further confirmed WHtR as a good indicator at discriminating those individuals at increased risk of hypertension, and in some cases, it is better than BMI, WC, and WHR.

## 1 Introduction

Hypertension is not only a common disease itself, but also one of the main causes for risk of cerebrovascular and cardiovascular diseases, such as stroke, metabolic syndromes, and coronary artery diseases.^[1–5]^ According to World Health Organization (WHO) Report in 2013, 1 billion individuals suffered from hypertension worldwide, and 9 million are deceased due to raised blood pressure annually.^[6]^ Moderate numbers of studies provided strong evidence that hypertension contributes markedly to the global burden of diseases.^[7–11]^ Although hypertension diagnosis seemed easier and cheaper than other cardiovascular diseases, no syndromes are reported by a number of people with high blood pressure. Additionally, some population is not engaged in annual physical examinations due to busy working, unlike to hospital, and self-feeling healthy and others. Therefore, the awareness, treatment, and control of hypertension are very low in some countries.^[12–20]^

Thus, applying some simple anthropometric adiposity indexes (AAI) in evaluating and predicting the risk groups of hypertension is valuable. Since obesity has a strong association with hypertension,^[21–24]^ 4 AAI are common to be used as risk evaluation indexes in many epidemiological studies,^[25–34]^ including body mass index (BMI), waist circumstance (WC), waist-to-hip ratio (WHR) and waist-to-height ratio (WHtR), all of which can be self-measured. Two meta-analytic reviews were published in 2008 and provided more supports for centralized obesity, especially WHtR, while BMI was the poorest discriminator for detecting cardiovascular risk factors in both male and female.^[35,36]^ Additionally, a robust association was observed among Asians compared to non-Asian populations.^[36]^

However, Lee et al^[35]^ only searched MEDLINE database up to 2006, and another study^[36]^ used the original data of 19 cross-sectional studies from 10 countries in the Asia-Pacific regions. A number of individual studies were reported in the last decade.^[37–44]^ Thus, we conducted an updated systematic review and meta-analysis and summarize literature evidence of association of hypertension risk with BMI, WC, WHR, and WHtR, as well as further evaluate sex-based and country-based difference for these associations. Our data in a prospective urban and rural epidemiology study in China (PURE-China) was added into meta-analyses as an individual study.

## 2 Methods

### 2.1 Searching strategies

All procedures of this study followed the guidelines of the preferred reporting items for systematic reviews and meta-analyses (PRISMA) statement.^[45]^ A systematic searching was conducted to identify the related articles in the following literature databases up to September 2018, including Cochrane Library (CENTRAL), PubMed, MEDLINE, EMBASE, Web of Science, WANFANG Data, China National Knowledge Infrastructure (CNKI), and SinoMed, and using the combinations of the following terms: (“body mass index” or “BMI”) and (“waist” or “waist circumference” or “WC”) and (“waist to hip ratio” or “waist-hip ratio” or “WHR” or “WHPR” or “waist; hip ratio”) and (“waist to height ratio” or “waist-height ratio” or “waist: height ratio” or “waist to stature ratio” or “waist-stature ratio” or “WHtR” or “WHTR” or “WSR” or “WHeiR”) and (“blood pressure” or “hypertension”). Corresponding Chinese terms with above-mentioned terms were used for searching in Chinese literature databases, such as CNKI, WANFANG Data, and SinoMed. All the bibliographical references found in target literature databases were imported into Endnote X8 for verifying eligibility checking. Each title and/or abstract was screened to evaluate its possible relevance after excluding duplicates. Full-text articles were downloaded for further review and eligibility determination if both titles and abstracts were not enough to make decision. All article-selecting were completed by 2 researchers (Deng GJ and Liu WD) independently, the senior researcher (Yin L) made final decision when any discrepancies were shown. Personal email contacts with authors were used to obtain data when needed data were not explicitly reported or not derived from data in the articles. Cross-referencing was also conducted to improve the study identification process.

### 2.2 Inclusive criteria

The inclusive criteria of article selection were described as follows:

(1) only original articles were considered, and editorials, comments or reviews were excluded;
(2) hypertension risk was evaluated in epidemiological studies;
(3) only adults were included (age≥18-year-old), but studies with older adults (age≥60-year-old) were excluded;
(4) odds ratio (OR) for the associations of hypertension risk with BMI, WC, WHR, and WHtR, and/or area under receiver operating characteristic curve (AUC) for prediction abilities of hypertension risk had to be reported in 1 study. Studies with lack of any one of the indexes above-mentioned were excluded.

### 2.3 Data extraction

If articles were regarded as eligible, at least 2 co-authors extracted the following data independently in a standardized manner and any disagreement was discussed and resolved in our research group, including author's name, publication year, country of study, study duration, study design, recruited participants (age, number, gender, BMI, WC, WHR, and WHtR), OR, and AUC with their 95% respective confidence interval (CI) for hypertension risk related to BMI, WC, WHR, and WHtR.

### 2.4 Literature quality assessment

The assessment for the quality and potential bias of the included articles were executed by 2 researchers independently using forms from Agency for Healthcare Research and Quality (AHRQ),^[46]^ which consists of 11 items scored 0 or 1. One score was counted if any item was answered “Yes”, while the score was 0 when any item was answered “No” or “Unclear”. The total score was calculated by adding all the scores of 11 items, and the quality level was determined as low if the total score≤3, medium if the score ranged from 4 to 7, and high if total score≥8.

### 2.5 General information of PURE-China

Details of PURE-China have been reported elsewhere.^[47,48]^ Based on 46,285 recruited participants, 1871 were excluded due to missing values of blood pressure, weight, height, WC, and hip circumference (HC) and 156 excluded due to implausible values for systolic blood pressure (SBP) (<70 or >260 mmHg), diastolic blood pressure (DBP) (<40 or >140 mmHg), weight (<30 or >130 kg), WC (>130 cm), and HC (<50 cm). Finally, 44,258 eligible subjects (18,174 male and 26,084 female) were included for the analyses.

Guided by 2010 Chinese guidelines of hypertension management,^[49]^ hypertension is defined if 1 of the following 3 criteria is fulfilled:

(1) taking antihypertensive drugs regularly;
(2) history of hypertension diagnosis;
(3) SBP≥140 mmHg and/or DBP≥90 mmHg. BMI was calculated as weight (kg) divided by height square (m^2^), WHR computed using WC (cm) divided by HC (cm), and WHtR using WC (cm) divided by height (cm).

### 2.6 Statistical analyses

Stata 12.0 was used for the meta-analyses. OR and AUC with their respective 95% CI for hypertension risk with 4 AAI (BMI, WC, WHR, WHtR) was defined as effect sizes. Heterogeneity was present if *P* value of *Q* test was typically ≤0.10. I^2^ statistic was used to evaluate the heterogeneity across all included studies. If studies were homogeneity, the pooled OR and pooled AUC were calculated by using a random effects model with DerSimonian and Laird method. If not, the fixed effect models on the Mantel-Haenszel method were applied.^[50–52]^*P* <.05 with 2-sided will be considered as statistical significance regarding the pooled results of all outcomes. Subgroup analyses based on gender were performed to compare potential variations among females and males. The potential publication bias was examined by constructing a “funnel plot”, and the Egger linear regression test was applied to test for asymmetry of funnel plots at 0.05 level for significance.^[53]^ In order to test for the robustness of the results, sensitivity analyses were conducted by deleting 1 study each time, which was considered as having little influence on the overall effect size if the point estimate of its “deleted” analysis always lay inside the 95% CI of the pooled statistic. Meta-regressions were used to examine the impact of moderator variables (including gender and country) on study effect sizes using regression-based techniques.^[54]^

The Statistical Analysis System (SAS 9.4 for Windows; SAS Institute Inc., Cary, NC) software was used for the statistical analyses of PURE-China. Only baseline data were used for analyses. Continuous variables were shown as the mean ± standard deviation (SD), and categorical variables as numbers (n) and percentages (%). The OR with 95% CI and AUC with 95% CI for hypertension risk in relation to BMI, WC, WHR, WHtR were computed using multivariate logistic regressions adjusted for age, sex (not for subgroup analyses by gender), education levels, alcohol use, smoking status, living location, levels of physical activities, as well as taking anti-diabetics drugs and lipid-lowering drugs. Subgroup analyses stratified by gender country and study design also were conducted.

## 3 Results

### 3.1 Systematic searching and article selection

**Figure 1:**
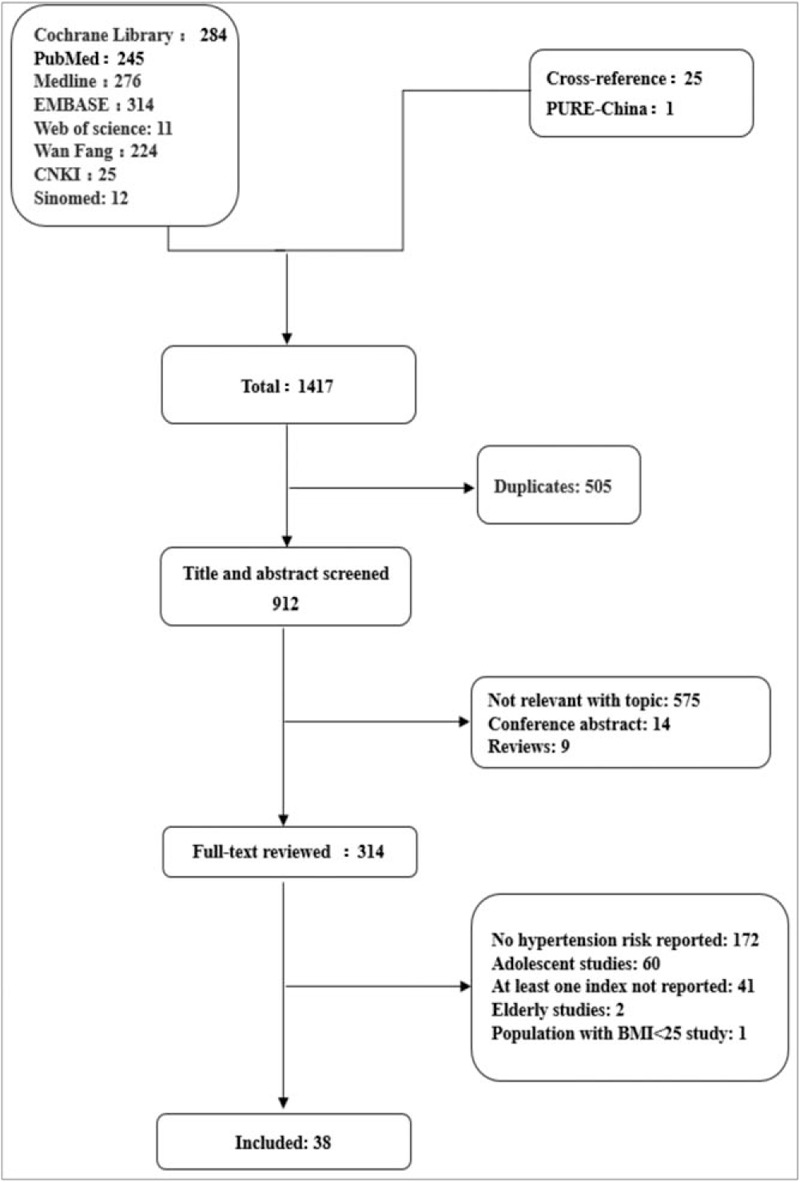
Flow diagram of the literature search process in meta-analysis. BMI = body mass index, PURE = prospective urban and rural epidemiology study.

The details of search strategy and included procedure were shown in Figure 1. Total of 1417 records was obtained from 8 above-mentioned literature databases and cross-referencing. PURE-China data were analyzed as an individual study. 505 duplicates were excluded. 912 titles and abstracts were screened for potential eligibility, among which 575 were deleted as irrelevant records with our topic, 14 were deleted as they were conference abstracts, and 9 were deleted as they were reviews. Furthermore, full-text reviewing of 314 records was performed, of which 216 were further excluded due to the following reasons: no hypertension risk reported (n = 172), adolescent studies (n = 60), at least 1 index not reported (n = 41), only older adults included (n = 2), only those with BMI <25 included (n = 1). Finally, a total of 309,585 individuals from 38 articles were included in this meta-analysis, including our PURE-China data.

The details of included studies were shown in Table 1. The included studies were published from 2002 to 2018, with sample size ranging from 180^[54]^ to 55,563.^[55]^ Only 6 studies had subjects less than 1000,^[40,42,54,56–58]^ and there were 6 studies with more than 10 thousand subjects,^[37,55,59–61]^ including PURE-China. According to AHRQ,^[46]^ the overall quality of the included studies was good with the average score 9.1, ranged from 7 to 10. 15 studies were scored at 10,^[39,41–43,62–71]^ including PURE-China, 13 studies at 9,^[40,44,56,58–61,72–77]^ 9 studies at 8,^[37,38,55,57,78–82]^ and 1 study at 7.^[54]^

**Table 1:**
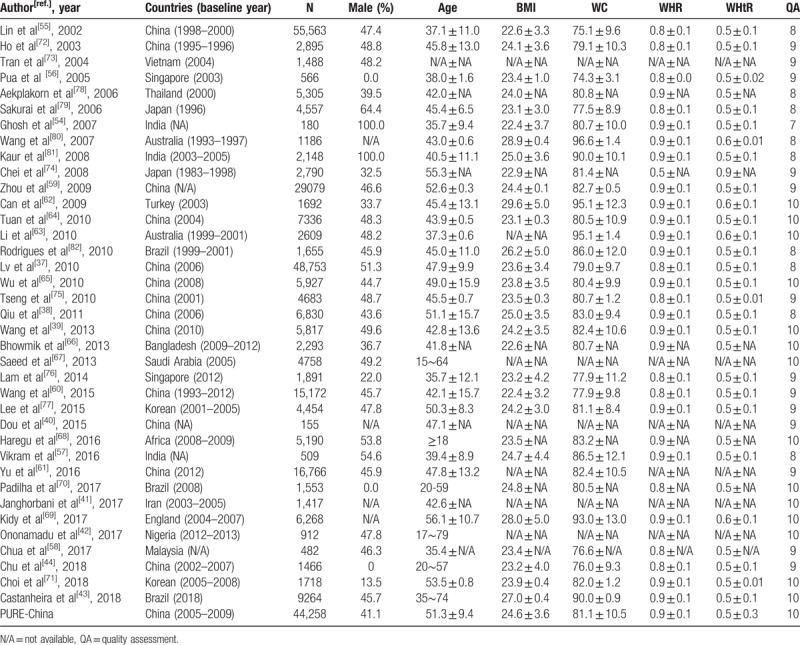
Characteristic of eligible studies in meta-analysis.

### 3.2 Results of PURE-China

Baseline characteristics of eligible participants in PURE-China were shown in Table 2. Total of 44,258 Chinese including 18,174 males and 26,084 females were included in this study, among which 19,100 (43.2%) were identified as patients suffering from hypertension. Mean age was similar among females and males (51.0 vs 51.6 years), but those with hypertension were much older than those without hypertension (54.6 vs 48.7 years). Additionally, 4 AAI were much higher among hypertension patients than normotensives, including BMI (25.6 vs 23.8 kg/m^2^), WC (84.4 vs 78.6 cm), WHR (0.88 vs 0.85), and WHtR (0.53 vs 0.49).

**Table 2:**
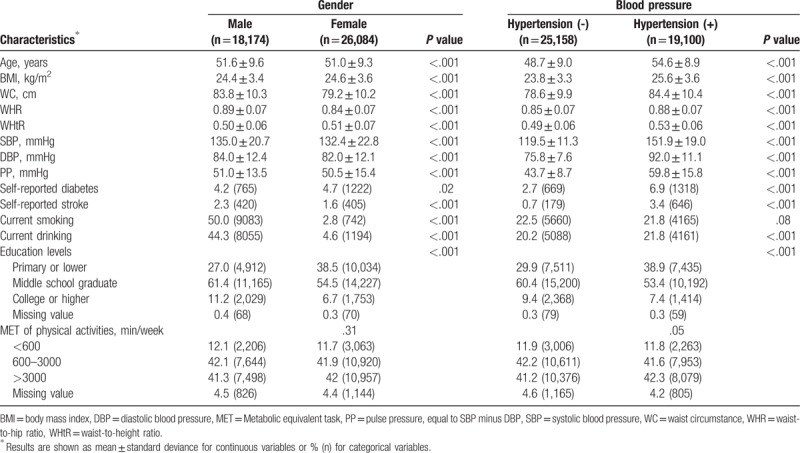
Characteristics of eligible participants in PURE-China.

OR and AUC and their respective 95% CI for hypertension risk according to various AAI in unadjusted and adjusted models were shown in Table 3. Significance was found for all associations of hypertension risk with 4 AAI in females, males, and both. The highest OR was observed for WHtR in both sexes (OR, 2.63; 95% CI, 2.54–2.71), women (OR, 2.76; 95% CI, 2.64–2.88), and men (OR, 2.51; 95% CI, 2.38–2.65) in unadjusted models. In adjusted models, the highest ORs were also observed for WHtR in both sexes (OR, 2.31; 95% CI, 2.23–2.40), as well as in women (OR, 2.15; 95% CI, 2.06–2.25) and in men (OR, 2.45; 95% CI, 2.31–2.60). The next was WHR (OR, 1.69; 95% CI, 1.64–1.75), and the 3^rd^ was BMI (OR, 1.17; 95% CI, 1.16–1.18). WC was found to be the poorest one (OR, 1.05; 95% CI, 1.05–1.06).

**Table 3:**
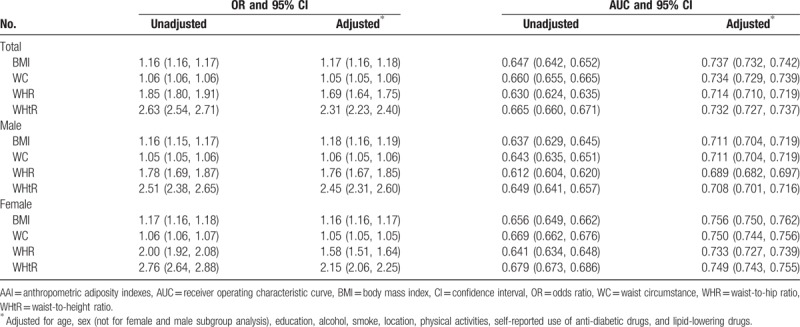
OR and AUC and their 95% CI for hypertension risk per various AAI.

Regarding prediction abilities of hypertension risk, WHtR was the strongest in unadjusted model (both sexes: AUC, 66.5%; 95% CI: 66.0%–67.1%; females: AUC, 67.9%; 95% CI, 67.3%–68.6%; males: AUC, 64.9%; 95% CI, 64.1%–65.7%). However, BMI showed strongest prediction abilities in adjusted models (AUC, 73.7%; 95% CI, 73.2%, 74.2%) among both sexes, in males (AUC, 71.1%; 95% CI, 70.4%–71.9%) and females (AUC, 75.6%; 95% CI, 75.0%–76.2%).

### 3.3 Meta-analysis results

#### 3.3.1 Overall ORs of meta-analyses

**Figure 2:**
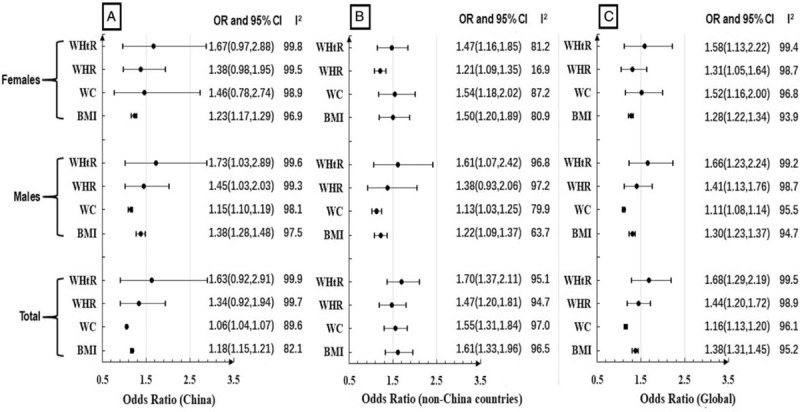
Summary ORs of BMI, WC, WHR, WHtR for hypertension risk in China (2A), non-China countries (2B) and global (2C). BMI = body mass index, CI = confidence interval, OR = odds ratio, WC = waist circumference, WHR = waist-to-hip ratio, WHtR = waist-to-height ratio.

The summary ORs of 4 AAI for hypertension risk in China, non-China countries and global were shown in Figure 2. Together with PURE-China, 10 articles^[40,54,61,67–69,76,80,81]^ reported ORs for the associations with hypertension risk, 8 articles^[38,54,59,66,74,75,81]^ reported ORs in men, 6 articles^[38,59,66,74,75]^ reported ORs in women. ORs from all countries were combined using meta-analysis methods and found WHtR was the highest OR (OR, 1.68; 95% CI, 1.29–2.19), followed WHR (OR, 1.44; 95% CI, 1.20–1. 72), the 3^rd^ for BMI (OR, 1.38; 95% CI, 1.31–1.45), and the lowest for WC (OR, 1.16; 95% CI, 1.13–1.20), but large heterogeneity was observed across individual studies (all I^2^ >95%). Publication bias was found for BMI (Egger test *P* = .003), WC (Egger test *P* = .001) and WHtR (Egger test *P* = .044), but not for WHR (Egger test *P* = .093). Further trim and fill analyses were conducted to obtain filled ORs for BMI (OR, 1.26; 95% CI, 1.19–1.33), WC (OR, 1.11; 95% CI, 1.07–1.15) and WHtR (OR, 1.68; 95% CI, 1.29–2.19), which still reached statistical significance. Sensitivity analyses were conducted to evaluate the stability of overall effect size, and no outliers were detected for overall effect sizes for WHR and WHtR. However, the study by Kaur 2008,^[81]^ Yu 2016^[61]^ and PURE-China were identified as outliers for BMI and WC. After deleting these 2 studies, larger OR were observed for both BMI (OR, 1.74; 95% CI, 1.46–2.06) and WC (OR, 1.61; 95% CI, 1.32–1.97).

Subgroup analyses were performed to evaluate the associations between 4 AAI and hypertension risk in China and non-China countries, as well as females and males, all of which were illustrated in Figure 2. These association strengths seemed similar in China and non-China countries (*P* for meta-regression = .59 for WHtR; .52 for WHR; .75 for WC; .95 for BMI). Additionally, no significant difference was observed for meta-regression based on gender among both China and non-China countries (*P* for meta-regression ≥.4 for the 4 indexes).

Further subgroup analyses were conducted to evaluate the associations between cross-sectional, retrospective cohort study and prospective cohort study. And found that BMI was the highest OR among prospective cohort study (OR, 1.24; 95% CI, 1.12–1.39) and retrospective cohort study (OR, 1.29; 95% CI, 1.21–1.37) respectively. However, it was WHtR with the highest OR among cross-sectional study (OR, 1.75; 95% CI, 1.41–2.17). Significant difference was observed for meta-regression based on study design (*P* for meta-regression<0.01 for the 4 indexes).

#### 3.3.2 Overall AUCs of meta-analyses

**Figure 3:**
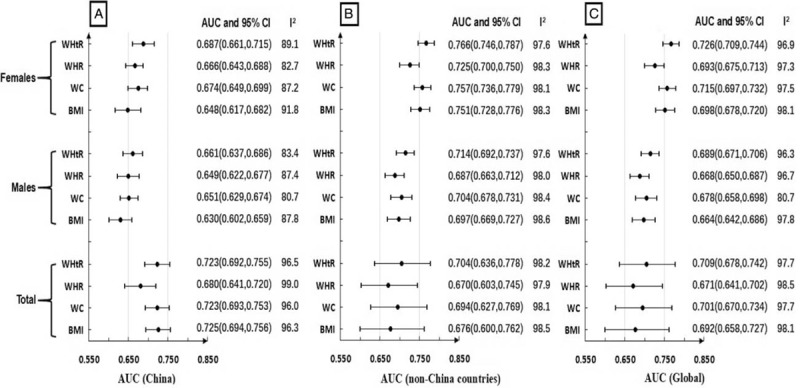
Summary AUCs of BMI, WC, WHR, WHtR for hypertension risk in China (3A), non-China countries (3B) and global (3C). AUC = receiver operating characteristic curve, BMI = body mass index, CI = confidence interval, WC = waist circumference, WHR = waist-to-hip ratio, WHtR = waist-to-height ratio.

Summary AUCs of 4 AAI for hypertension risk was illustrated in Figure 3. Together with PURE-China study, a total of 31 articles^[37–39,41–44,55–66,69–73,75–79,82]^ reported AUCs, including 13 articles^[37–39,44,55,59–61,64,65,72,75]^ from China, and 18 articles^[41–43,56–58,62,63,66,69–71,73,76–79,82]^ from other countries outside of China. In random effects models of meta-analysis, WHtR had the strongest prediction abilities of hypertension risk in both sexes (AUC, 70.9%; 95% CI: 67.8%–74.2%), whatever males (AUC, 68.9%; 95% CI: 67.1%–70.6%) and females (AUC, 72.6%; 95% CI: 70.9%–74.4%). Prediction abilities were higher among China studies than other countries (*P* for meta-regression <.01 for the 4 indexes). Large heterogeneity was observed for all meta-analyses for AUCs (all I^2^>80%). No outliers were identified in sensitivity analyses for WHtR, WHR, WC, and BMI, and no publication bias was found (all Egger test *P* >.10). Trim and fill analyses were conducted to evaluate prediction abilities after filling “missing studies”, filled AUC continued to show original prediction abilities for all 4 AAI.

Subgroup analyses based on gender and China and non-China countries were also conducted, which were illustrated in Figure 3. Significant difference was observed for meta-regression between China and non-China countries (*P* <.01 for the 4 indexes). Significant difference was observed for meta-regression between males and females in China for BMI (*P* = .03), WC (*P* = .04) and WHtR (*P* = .02). However, no significant difference was observed for meta-regression between males and females among non-China countries (*P* >.2 for the 4 indexes).

Further subgroup analyses were conducted to evaluate the associations between cross-sectional, retrospective cohort study, and prospective cohort study. WHtR had the strongest prediction abilities of hypertension risk among prospective cohort study (AUC, 64.4%; 95% CI, 60.3%–68.7%), cross-sectional study (AUC, 70.4%; 95% CI, 68.8%–72.1%) and retrospective cohort study (AUC, 74.5%; 95% CI, 69.0%–71.9%) respectively. Significant difference was observed for meta-regression based on study design (*P* for meta-regression <.01 for the 4 indexes).

## 4 Discussions

Together with PURE-China study, 38 articles involving 309,585 participants were identified to evaluate the associations of hypertension risk with 4 AAI, including BMI, WC, WHR, and WHtR using systematic review and meta-analysis strategies. Our results further confirmed the positive associations between hypertension risk and these AAI. Among the 4 AAI, WHtR has the strongest prediction ability for hypertension risk, irrespective of the gender, though large heterogeneity and publication bias were observed across the included studies. Further sensitivity analyses and trim and fill analyses did not alter the respective prediction abilities.

Our meta-analyses updated the results of 2 previous meta-analytic reviews^[35,36]^ and further confirmed that WHtR had the highest pooled AUC and OR among the global countries. WHO report also recommended that WC, WHR, WHtR were superior to BMI in predicting CVD risk respectively.^[83]^ Most studies provided more supports for central adiposity in predicting CVD risk including hypertension risk, especially WHtR;^[22,84–86]^ however, some studies suggested that WC is the best indicator for reflecting the associations between obesity and hypertension risk.^[24,87]^ Adjusted results from PURE-China showed that WHtR had the strongest association with hypertension risk, while BMI had the strongest prediction ability for hypertension, which might be related to other valuable confounders, such as alcohol use, smoking status, physical activities, and medication use, though AUC of WHtR was the best in unadjusted models. Nonetheless, several studies^[37–44,54–58,60,62–65,67,70–73,77–79,82]^ did not report adjusted ORs and AUCs. We combined the effect sizes from 10 studies^[59,61,66,68,69,74–76,80,81]^ with adjusted ORs, and found both BMI and WHtR (both OR, 1.41) were superior to WC (OR, 1.20) and WHR (OR, 1.28). We also combined effect sizes from 4 studies^[59,61,69,76]^ with adjusted AUCs and found that the prediction ability of BMI, WC, and WHtR were almost the same (all AUC, 74%–75%), which little superior to WHR (AUC, 72.2%). Hence, more studies are needed to confirm this variation, and hitherto, BMI and WC are not excluded while predicting the risk of hypertension.

Similar to previous studies,^[35–37]^ significant heterogeneity among females and males was observed when discriminating hypertension risk, and higher combined AUCs were found among females than males, which indicated that the hypertension risk was estimated rather precisely in women. Furthermore, except for WC, the association of hypertension risk was stronger in men than women, although this correlation variation was not confirmed in meta-regression with respect to sex. Additionally, the difference in discrimination abilities for hypertension risk in China and other countries are notable. According to OR, WHtR is the best predictor for both Chinese population and other ethnic groups. When considering about AUC, while the best predictors are BMI and WHtR for China and non-China countries respectively. And current evidence indicated that the strength of the association between the anthropometric measures with hypertension risk is higher in other countries than China, irrespective of indexes. Central adiposity has been emphasized by a number of studies, particularly for Asian populations who may have a ‘normal’ BMI along with disproportionately large WC.^[36,37]^ However, BMI showed the strongest prediction abilities in adjusted models in our PURE-China study, in either females or males, or both sexes.

Our study has specific strengths and limitations. A major strength is the application of systematic review strategies and comprehensive evaluation of the associations between adiposity measures and hypertension risk from available data, despite large heterogeneity and publication bias were observed. First, major limitations are related to limitations of the data provided by the individual studies. As a result, the risk estimation may be less accurate if individual-level data were not been available. Some studies were excluded due to no complete data used for meta-analyses, even if we contacted with authors via emails.^[88–91]^ Second, most of studies included in our meta-analyses were observational studies, which have potential methodological limitations to detect causality between exposure and outcome. Third, 3 studies including our PURE-China were defined as outliers when assessing the stability of effect sizes of BMI and WC. Additionally, potential publication bias was detected using Egger tests, though Begg and Mazumdar rank correlation test not. Finally, although 8 databases were searched for the reviews and extensive checks for completeness by cross-referencing were employed, we cannot promise that a relevant study might be missed.

## 5 Conclusions

Despite these limitations, our systematic review and meta-analyses summarize the available studies so far and provide a comprehensive picture for the associations between hypertension risk and 4 anthropometric measures. The magnitude of these association was partly similar among Chinese and non-Chinese populations. WHtR was confirmed as a good indicator at discriminating those individuals at increased risk of hypertension.

## Acknowledgments

Besides co-authors listed in this study, we would like to thank Ononamadu, CJ from Department of Biochemistry and Forensic science, Nigerian police academy, who share the data we need with us. In addition, we would like to thank those who supported our study and what they did with our sincere gratitude, especially for site coordinators, physicians, nurses, questionnaire interviewers, and laboratory personnel in all participating centers and communities, included China Coordination Center Beijing Office: Lisheng Liu, Hongye Zhang, Jian Bo, Jian Li, Kean Wang, Xiaoru Cheng, Xixin Hou, Xingyu Wang, Xuan Jia, Yi Sun, Yang Wang, Xiaoyun Liu; Jishuitan Hospital, Beijing: Di Chen, Dong Li, Hui Jin, Jiwen Tian; Center for Disease Control & Prevention, Shunyi District, Beijing: Yindong Li, Kai You, Changqing Li, Songjian Zhang,Wenlong Cheng,Hongye Zhang; Hospital of Traditional Chinese Medicine, Shijingshan District, Beijing: Honghong Li, Qiang Zhou, Xu Xu, Yanhong Sun, Jinling Di, Jianquan Wu, Mei Wang; Bayannaoer Center for Disease Control & Prevention, Inner Mongolia: Minzhi Cao, Shiying Zhang, Aiying Han; Center for Disease Control & Prevention, Wujin District, Changzhou City, Jiangsu Province: Jianxin Zhou, Yihong Zhou, Deren Qiang, Jianfang Wu, Zhaowei Li, Jing Qin, Suyi Shi, Zhihua Fan, Alin Qian, Lingyun Pan, Minrui Xu, Yibing Cui; Jiangsu provincial hospital:Jun Li,Yongzhen Mo, Center for Disease Control & Prevention, Jiangsu Province: Quanyong Xiang; Ye Cao, Jiangxinzhou Community Health Service Center, Nanjing City, Jiangsu Province: Zhenzhen Qian, Zhengrong Liu; Health Service Center, Nanhu District, Nanjing City, Jiangsu Province: Xiangrong He; Changlin Dong, Ming Wan; Xiaohang Hospital, Nanjing City, Jiangsu Province: Jinhua Tang; Center for Disease Control & Prevention, Nanchang County, Jiangxi Province: Rensheng Lei, Lihua Hu, Shuwei Xiong; Qingshanhu Community Health Service Center, Nanchang City, Jiangxi Province: Ning Li, Xincheng Tang, Dan Zou, Qilu Gan, Shuli Ye; Shenyang 242 Hospital, Liaoning Province: Yu Liu; Health Center, Daxing District, Shenyang City, Liaoning Province: Minfan Fu, Qiuyuan Wang; RedCross Hospital, Shenyang City, Liaoning Province: Baoxia Guo, Huilian Feng, Xiaojie Xing; Center for Disease Control & Prevention, Xining City, Qinghai Province: Yuqing Yang, Wenqiang Xu, Haibin Ma, Yali Wang; Huizu Hospital, Xining City, Qinghai Province: Youzhu Yang, Xiaolan Ma, Yan Hai, Zhe Xie, Yuanting Ma; Huaxi Hospital, Chengdu City, Sichuan Province: Xiaoyang Liao, Qian Zhao,Chuan Zou; Jianshe Road Community Health Service Center, Chengdu City, Sichuan Province: Guofan Xu, Jiankang Liu; Health Center, Dayicaichang Town, Sichuan Province: Xiaolin Zhang, Wenqing Deng; Cardiovascular Disease Research Institute, Shandong Province: Fanghong Lu, Hua Zhang, Shangwen Sun, Yingxin Zhao, Zhendong Liu, Falian Sun; Jingle County Hospital, Shanxi Province: Yinsheng Wu, Guoqin Liu; Balingqiao CommunityHealthServiceStation, Xinghualing District, Taiyuan City, Shanxi Province: Yan Hou, Junying Wang, Hua Wei; Electronic Science and Technology University Hospital, Xi’an City, Shaanxi Province: Xiaoxia Li, Yahong Zhi, Tianlu Liu; Guanshan Town Hospital, Yanliang District, Xi’an City, Shaanxi Province: Peng Zhang; Center for Disease Control & Prevention, Hetian City, Xinjiang Province: Ayoufu Aideer Aili, Mitiwula, Reshalaiti, Hui Wang; Health Center, Damenglong Town, Xishuangbanna Prefecture, Yunnan Province: Qiyun Wang, Jinkui Yang, Kehua Li; Center for Disease Control & Prevention, Mengla County, Xishuangbanna Prefecture, Yunnan Province: Huaxing Liu, Chunmei Liu; Center for Disease Control & Prevention, Yunnan Province: Yize Xiao.

## Author contributions

**Conceptualization:** Guijuan Deng, Lu Yin.

**Data curation:** Guijuan Deng, Xiaoyun Liu, Juntao Ma, Bo Hu.

**Formal analysis:** Guijuan Deng.

**Funding acquisition:** Wei Li.

**Investigation:** Guijuan Deng, Lu Yin, Quanyong Xiang, Zhenzhen Qian, Hui Chen, Bo Hu.

**Methodology:** Guijuan Deng, Lu Yin.

**Project administration:** Lu Yin.

**Resources:** Quanyong Xiang, Zhenzhen Qian, Juntao Ma.

**Software:** Guijuan Deng, Weida Liu, Yang Wang.

**Supervision:** Lu Yin, Quanyong Xiang, Wei Li, Yu Jiang.

**Validation:** Weida Liu, Zhenzhen Qian.

**Visualization:** Guijuan Deng.

**Writing – original draft:** Guijuan Deng.

**Writing – review & editing:** Lu Yin, Yu Jiang.

## References

[1] Zhang Q Mahapatra T Huang F Association between anthropometric measures and indicators for hypertension control among Kazakh-Chinese hypertension patients in Xinjiang, China: results from a cross-sectional study. PLoS One 2017;12:1–3. 10.1371/journal.pone.0170959 PMC5271364 28129402

[2] Dong B Wang Z Wang HJ Associations between adiposity indicators and elevated blood pressure among Chinese children and adolescents. J Hum Hypertens 2015;29:236–40. 25339297 10.1038/jhh.2014.95

[3] Redon J Different strategies from monotherapies to dual or triple fixed dose combination therapies to achieve blood pressure goals: a summary of a satellite symposium from the European Society of Hypertension, June 13-16, 2014 Athens, Greece. Introduction. High Blood Press Cardiovasc Prev 2015;22 suppl 1:S3–4. 25929685 10.1007/s40292-015-0095-2

[4] Mancia G Introduction to a compendium on hypertension. Circ Res 2015;116:923–4. 25767280 10.1161/CIRCRESAHA.115.305755

[5] Chen SC Lo TC Chang JH Variations in aging, gender, menopause, and obesity and their effects on hypertension in taiwan. Int J Hypertens 2014;2014:1–7. 10.1155/2014/515297 PMC4243128 25436143

[6] WHO: World Health Organization. A global brief on Hypertension: Silent killer, global public healthrisis. http://www.who.int: World Health Organization; 2013.

[7] Lackland DT Weber MA Global burden of cardiovascular disease and stroke: hypertension at the core. Can J Cardiol 2015;31:569–71. 25795106 10.1016/j.cjca.2015.01.009

[8] Forouzanfar MH Liu P Roth GA Global Burden of Hypertension and Systolic Blood Pressure of at Least 110 to 115 mm Hg, 1990-2015. JAMA 2017;317:165–82. 28097354 10.1001/jama.2016.19043

[9] Mensah GA The global burden of hypertension: good news and bad news. Cardiol Clin 2002;20:181–5. 12119794 10.1016/s0733-8651(02)00002-4

[10] Brody AM Kumar VA Levy PD Hot topic: global burden of treating hypertension-what is the role of the emergency department. Curr Hypertens Rep 2017;19:1–4. 28176250 10.1007/s11906-017-0707-4

[11] Tadic M Cuspidi C Hering D Hypertension and cognitive dysfunction in elderly: blood pressure management for this global burden. BMC Cardiovasc Disord 2016;16:1–9. 27809779 10.1186/s12872-016-0386-0 PMC5093934

[12] Gupta R Kaur M Islam S Association of household wealth index, educational status, and social capital with hypertension awareness, treatment, and control in South Asia. Am J Hypertens 2017;30:373–81. 28096145 10.1093/ajh/hpw169

[13] Gebrihet TA Mesgna KH Gebregiorgis YS Awareness, treatment, and control of hypertension is low among adults in Aksum town, northern Ethiopia: a sequential quantitative-qualitative study. PLoS One 2017;12:1–6. 10.1371/journal.pone.0176904 PMC5425176 28489865

[14] Sanuade OA Awuah RB Kushitor M Hypertension awareness, treatment and control in Ghana: a cross-sectional study. Ethn Health 2018;2:1–5. 10.1080/13557858.2018.1439898 29448808

[15] Khanal MK Dhungana RR Bhandari P Prevalence, associated factors, awareness, treatment, and control of hypertension: findings from a cross sectional study conducted as a part of a community based intervention trial in Surkhet, mid-western region of Nepal. PLoS One 2017;12:1–20. 10.1371/journal.pone.0185806 PMC5628876 28982159

[16] Yusufali AM Khatib R Islam S Prevalence, awareness, treatment and control of hypertension in four Middle East countries. J Hypertens 2017;35:1457–64. 28486270 10.1097/HJH.0000000000001326

[17] Lemogoum D Van de Borne P Lele CEB Prevalence, awareness, treatment, and control of hypertension among rural and urban dwellers of the far north region of Cameroon. J Hypertens 2018;36:159–68. 29210863 10.1097/HJH.0000000000001513

[18] Wang ZQ Zhao YF Yang J Rate of prevalence, awareness, treatment and control of hypertension among women at reproductive age in China in 2013. Zhonghua Yu Fang Yi Xue Za Zhi 2017;51:1086–90. 29262489 10.3760/cma.j.issn.0253-9624.2017.12.007

[19] Herrera-Anazco P Pacheco-Mendoza J Valenzuela-Rodriguez G Self-knowledge, adherence to treatment, and control of arterial hypertension in peru: a narrative review. Rev Peru Med Exp Salud Publica 2017;34:497–504. 29267775 10.17843/rpmesp.2017.343.2622

[20] Shafi ST Shafi T A survey of hypertension prevalence, awareness, treatment, and control in health screening camps of rural central Punjab, Pakistan. J Epidemiol Glob Health 2017;7:135–40. 28188121 10.1016/j.jegh.2017.01.001 PMC7320434

[21] Huang KC Lin WY Lee LT Four anthropometric indices and cardiovascular risk factors in Taiwan. Int J Obes Relat Metab Disord 2002;26:1060–8. 12119571 10.1038/sj.ijo.0802047

[22] Kazempour-Ardebili S Ramezankhani A Eslami A Metabolic mediators of the impact of general and central adiposity measures on cardiovascular disease and mortality risks in older adults: Tehran lipid and glucose study. Geriatr Gerontol Int 2017;17:2017–24. 28349639 10.1111/ggi.13015

[23] Nguyen T Lau DC The obesity epidemic and its impact on hypertension. Can J Cardiol 2012;28:326–33. 22595448 10.1016/j.cjca.2012.01.001

[24] Yasien N Jarrah S Petro-Nustas W Obesity indices and their relationship to cardiovascular risk factors in young adult group. J Bahrain Med Soc 2010;22:133–7.

[25] Wang S Ma W Yuan Z Association between obesity indices and type 2 diabetes mellitus among middle-aged and elderly people in Jinan, China: a cross-sectional study. BMJ Open 2016;6:1–9. 10.1136/bmjopen-2016-012742 PMC5129047 27810975

[26] Sattar N Tan CE Han TS Associations of indices of adiposity with atherogenic lipoprotein subfractions. Int J Obesity 1998;22:432–9. 10.1038/sj.ijo.0800604 9622340

[27] Liu Y Tong G Tong W Can body mass index, waist circumference, waist-hip ratio and waist-height ratio predict the presence of multiple metabolic risk factors in Chinese subjects. BMC Public Health 2011;11:1–0. 21226967 10.1186/1471-2458-11-35 PMC3032682

[28] Hori A Nanri A Sakamoto N Comparison of body mass index, waist circumference, and waist-to-height ratio for predicting the clustering of cardiometabolic risk factors by age in Japanese workers—Japan epidemiology collaboration on occupational health study. Circ J 2014;78:1160–8. 24662439 10.1253/circj.cj-13-1067

[29] Farrag A El Hag A Hassan AMR Correlations between various obesity parameters and coronary artery disease severity. Eur Heart J 2011;32:1–301.

[30] Jeong SK Seo MW Kim YH Does waist indicate dyslipidemia better than BMI in Korean adult population. J Korean Med Sci 2005;20:7–12. 15716594 10.3346/jkms.2005.20.1.7 PMC2808579

[31] Tian S Zhang X Xu Y Feasibility of body roundness index for identifying a clustering of cardiometabolic abnormalities compared to BMI, waist circumference and other anthropometric indices: the China Health and Nutrition Survey, 2008 to 2009. Medicine (Baltimore) 2016;95:1–0. 10.1097/MD.0000000000004642 PMC5400331 27559964

[32] Wessel TR Arant CB Olson MB Relationship of physical fitness vs body mass index with coronary artery disease and cardiovascular events in women. JAMA 2004;292:1179–87. 15353530 10.1001/jama.292.10.1179

[33] Blaslov K Bulum T Duvnjak L Waist-to-height ratio is independently associated with chronic kidney disease in overweight type 2 diabetic patients. Endocr Res 2015;40:194–8. 25536141 10.3109/07435800.2014.987868

[34] Savva SC Tornaritis M Savva ME Waist circumference and waist-to-height ratio are better predictors of cardiovascular disease risk factors in children than body mass index. Int J Obes Relat Metab Disord 2000;24:1453–8. 11126342 10.1038/sj.ijo.0801401

[35] Lee CM Huxley RR Wildman RP Indices of abdominal obesity are better discriminators of cardiovascular risk factors than BMI: a meta-analysis. J Clin Epidemiol 2008;61:646–53. 18359190 10.1016/j.jclinepi.2007.08.012

[36] Obesity in Asia Collaboration Is central obesity a better discriminator of the risk of hypertension than body mass index in ethnically diverse populations. J Hypertens 2008;26:169–77. 18192826 10.1097/HJH.0b013e3282f16ad3

[37] Lv XZ Zhan SY Prediction of hypertension using anthropometric indices in adult aged 35∼74, in Taiwan. Chin J Dis Control Prev 2010;05:372–5.

[38] Qiu X Zheng C Du J Correlation of obesity with hypertension and diabetes. Chin J Cardiovasc Med 2011;2:93–6.

[39] Wang H Han Y Chen T Screening for optimal obesity index and its cutoff value for prediction of hypertension. Chin J Pub Heal 2013;29:1752–4.

[40] Dou C Hai X He J Correlation between physical characteristics and blood pressure of adult monks in Shigatse District in Tibet. Chin J Anat 2015;6:733–6.

[41] Janghorbani M Aminorroaya A Amini M Comparison of different obesity indices for predicting incident hypertension. High Blood Press Cardiovasc Prev 2017;24:157–66. 28160265 10.1007/s40292-017-0186-3

[42] Ononamadu CJ Ezekwesili CN Onyeukwu OF Comparative analysis of anthropometric indices of obesity as correlates and potential predictors of risk for hypertension and prehypertension in a population in Nigeria. Cardiovasc J Afr 2017;28:92–9. 27701484 10.5830/CVJA-2016-061 PMC5488060

[43] Castanheira M Chor D Braga JU Predicting cardiometabolic disturbances from waist-to-height ratio: findings from the Brazilian Longitudinal Study of Adult Health (ELSA-Brasil) baseline. Public Health Nutr 2018;21:1028–35. 29310740 10.1017/S136898001700338X PMC10261066

[44] Chu FL Jeng C Lowered obesity indicator cutoff points more effectively predict 5-year incidence of hypertension in premenopausal women. Int J Qual Health Care 2018 1–6. 10.1093/intqhc/mzy183 30165634

[45] Moher D Liberati A Tetzlaff J Group P Preferred reporting items for systematic reviews and meta-analyses: the PRISMA statement. Int J Surg 2010;8:336–41. 20171303 10.1016/j.ijsu.2010.02.007

[46] Rostom A Dubé C Cranney A Cranney A Celiac Disease. Rockville (MD): Agency for Healthcare Research and Quality (US). 2004 Sep. (Evidence Reports/Technology Assessments, No. 104.) Appendix D. Available at: https://www.ncbi.nlm.nih.gov/books/NBK35149/.

[47] Li W Gu H Teo KK Hypertension prevalence, awareness, treatment, and control in 115 rural and urban communities involving 47 000 people from China. J Hypertens 2016;34:39–46. 26630211 10.1097/HJH.0000000000000745

[48] Peng Y Li W Wang Y The cut-off point and boundary values of waist-to-height ratio as an indicator for cardiovascular risk factors in Chinese adults from the PURE study. PLoS One 2015;10:1–2. 10.1371/journal.pone.0144539 PMC4671670 26642201

[49] Writing Group of 2020 Chinese Guidelines for the Management of Hypertension. 2010 Guidelines for the prevention and treatment of hypertension in China; 2010:1–38. Available at: http://www.nccd.org.cn/UploadFile/201504/20150418172824476476.pdf.

[50] Dersimonian R Meta-analysis in clinical trials. Control Clin Trials 1986;7:177–88. 3802833 10.1016/0197-2456(86)90046-2

[51] Normand SL Meta-analysis: formulating, evaluating, combining, and reporting. Stat Med 1999;18:321–59. 10070677 10.1002/(sici)1097-0258(19990215)18:3<321::aid-sim28>3.0.co;2-p

[52] Lipsey MW Wilson DB Practical meta-analysis. Vol. 49. Thousand Oaks, CA: Sage; 2001.

[53] Rothstein HR Sutton AJ Borenstein M Publication Bias in Meta-Analysis: Prevention, Assessment and Adjustments[M]// Reports from the UK National Ecosystem Assessment Follow-on Phase 2014

[54] Ghosh JR Bandyopadhyay AR Comparative evaluation of obesity measures: relationship with blood pressures and hypertension. Singapore Med J 2007;48:232–5. 17342293

[55] Lin W Lee L Chen C Optimal cut-off values for obesity: using simple anthropometric indices to predict cardiovascular risk factors in Taiwan. Int J Obesity 2002;26:1232–8. 10.1038/sj.ijo.0802040 12187401

[56] Pua Y-H Ong P-H Anthropometric indices as screening tools for cardiovascular risk factors in Singaporean women. Asia Pac J Clin Nutr 2005;14:74–9. 15734711

[57] Vikram NK Latifi AN Misra A Waist-to-height ratio compared to standard obesity measures as predictor of cardiometabolic risk factors in Asian Indians in North India. Metab Syndr Relat Disord 2016;14:492–9. 27740885 10.1089/met.2016.0041

[58] Chua EY Zalilah MS Haemamalar K Obesity indices predict hypertension among indigenous adults in Krau Wildlife Reserve, Peninsular Malaysia. J Health Popul Nutr 2017;36:1–7. 28545536 10.1186/s41043-017-0102-4 PMC5445453

[59] Zhou Z Hu D Chen J Association between obesity indices and blood pressure or hypertension: which index is the best. Public Health Nutr 2009;12:1061–71. 18778533 10.1017/S1368980008003601

[60] Wang S Liu Y Li F A novel quantitative body shape score for detecting association between obesity and hypertension in China. BMC Public Health 2015;15:1–9. 25595192 10.1186/s12889-014-1334-5 PMC4308906

[61] Yu J Tao Y Tao Y Optimal cut-off of obesity indices to predict cardiovascular disease risk factors and metabolic syndrome among adults in Northeast China. BMC Public Health 2016;16:1–7. 27737656 10.1186/s12889-016-3694-5 PMC5062901

[62] Can AS Bersot TP Gonen M Anthropometric indices and their relationship with cardiometabolic risk factors in a sample of Turkish adults. Public Health Nutr 2009;12:538–46. 18489811 10.1017/S1368980008002474

[63] Li M McDermott RA Using anthropometric indices to predict cardio-metabolic risk factors in Australian indigenous populations. Diabetes Res Clin Pract 2010;87:401–6. 20034692 10.1016/j.diabres.2009.12.004

[64] Tuan NT Adair LS Stevens J Prediction of hypertension by different anthropometric indices in adults: the change in estimate approach. Public Health Nutr 2010;13:639–46. 19758482 10.1017/S1368980009991479 PMC2855402

[65] Wu H Zhu Q Gu J A cross-sectional study on the relationship between nthropometric indices and blood pressures among the residents of Pudong new area of Shanghai. Fudan Univ J Med Sci 2010;37:401–8.

[66] Bhowmik B Munir SB Diep LM Anthropometric indicators of obesity for identifying cardiometabolic risk factors in a rural Bangladeshi population. J Diabetes Investig 2013;4:361–8. 10.1111/jdi.12053 PMC4020230 24843680

[67] Saeed AA Al-Hamdan NA Anthropometric risk factors and predictors of hypertension among Saudi adult population—a national survey. J Epidemiol Glob Health 2013;3:197–204. 24206791 10.1016/j.jegh.2013.08.004 PMC7320412

[68] Haregu TN Oti S Egondi T Measurement of overweight and obesity an urban slum setting in sub-Saharan Africa: a comparison of four anthropometric indices. BMC Obes 2016;3:1–8. 27833755 10.1186/s40608-016-0126-0 PMC5100227

[69] Kidy FF Dhalwani N Harrington DM Associations between anthropometric measurements and cardiometabolic risk factors in white European and South Asian adults in the United Kingdom. Mayo Clin Proc 2017;92:925–33. 28578782 10.1016/j.mayocp.2017.02.009

[70] Padilha BM Diniz AS Ferreira HS Anthropometric predictors of hypertension in afro-descendant women. Sci Med 2017;27:1–9.

[71] Choi JR Ahn SV Kim JY Comparison of various anthropometric indices for the identification of a predictor of incident hypertension: the ARIRANG study. J Hum Hypertens 2018;32:294–300. 29581555 10.1038/s41371-018-0043-4

[72] Ho SY Lam TH Janus ED Hong Kong Cardiovascular Risk Factor Prevalence Study Steering C Waist to stature ratio is more strongly associated with cardiovascular risk factors than other simple anthropometric indices. Ann Epidemiol 2003;13:683–91. 14599732 10.1016/s1047-2797(03)00067-x

[73] Tran C Assessment of the prevalence of obesity and related risk factors in Vietnamese adults living in urban areas of Ho Chi Minh City, Vietnam[D]: Medical Science Faculty of Health, University of New Castle; 2004.

[74] Chei CL Iso H Yamagishi K Body fat distribution and the risk of hypertension and diabetes among Japanese men and women. Hypertens Res 2008;31:851–7. 18712039 10.1291/hypres.31.851

[75] Tseng CH Chong CK Chan TT Optimal anthropometric factor cutoffs for hyperglycemia, hypertension and dyslipidemia for the Taiwanese population. Atherosclerosis 2010;210:585–9. 20053403 10.1016/j.atherosclerosis.2009.12.015

[76] Lam BC Koh GC Chen C Comparison of body mass index (BMI), body adiposity index (BAI), waist circumference (WC), waist-to-hip ratio (WHR) and waist-to-height ratio (WHtR) as predictors of cardiovascular disease risk factors in an adult population in Singapore. PLoS One 2014;10:1–5. 10.1371/journal.pone.0122985 PMC4400161 25880905

[77] Lee JW Lim NK Baek TH Anthropometric indices as predictors of hypertension among men and women aged 40-69 years in the Korean population: the Korean Genome and Epidemiology Study. BMC Public Health 2015;15:1–7. 25886025 10.1186/s12889-015-1471-5 PMC4332746

[78] Aekplakorn W Kosulwat V Suriyawongpaisal P Obesity indices and cardiovascular risk factors in Thai adults. Int J Obes (Lond) 2006;30:1782–90. 16619055 10.1038/sj.ijo.0803346

[79] Sakurai M Miura K Takamura T Gender differences in the association between anthropometric indices of obesity and blood pressure in Japanese. Hypertens Res 2006;29:75–80. 16755140 10.1291/hypres.29.75

[80] Wang Z Rowley K Wang Z Anthropometric indices and their relationship with diabetes, hypertension and dyslipidemia in Australian Aboriginal people and Torres Strait Islanders. Eur J Cardiovasc Prev Rehabil 2007;14:172–8. 17446794 10.1097/01.hjr.0000220580.34763.fb

[81] Kaur P Radhakrishnan E Sankarasubbaiyan S A comparison of anthropometric indices for predicting hypertension and type 2 diabetes in a male industrial population of Chennai, South India. Ethn Dis 2008;18:31–6. 18447096

[82] Rodrigues SL Baldo MP Mill JG Association of waist-stature ratio with hypertension and metabolic syndrome: population-based study. Arq Bras Cardiol 2010;95:186–91. 20549135 10.1590/s0066-782x2010005000073

[83] World Health Organization. Waist Circumference and Waist-Hip Ratio Report of a WHO Expert Consultation: Report of a WHO Expert Consultation. WHO Library Cataloguing-in-Publication Data: World Health Organization;8–11 December 2008.

[84] Hsu HS Liu CS Pi-Sunyer FX The associations of different measurements of obesity with cardiovascular risk factors in Chinese. Eur J Clin Invest 2011;41:393–404. 21114491 10.1111/j.1365-2362.2010.02421.x

[85] Cai L Liu A Zhang Y Waist-to-height ratio and cardiovascular risk factors among Chinese adults in Beijing. PLoS One 2013;8:1–6. 10.1371/journal.pone.0069298 PMC3709905 23874938

[86] Ashwell M Gibson S Waist-to-height ratio as an indicator of ’early health risk’: simpler and more predictive than using a ’matrix’ based on BMI and waist circumference. BMJ Open 2016;6:1–7. 10.1136/bmjopen-2015-010159 PMC4800150 26975935

[87] Zhong Y Tian ZW Liu BR Association between adiposity indicators and hypertension over 35 years old. Zhong Guo Quan Ke Yi Xue 2012;15:4064–7.

[88] Meseri R Ucku R Unal B Waist:height ratio: a superior index in estimating cardiovascular risks in Turkish adults. Public Health Nutr 2014;17:2246–52. 24103435 10.1017/S136898001300267X PMC10282623

[89] Patel SA Deepa M Shivashankar R Comparison of multiple obesity indices for cardiovascular disease risk classification in South Asian adults: the CARRS Study. PLoS One 2017;12:1–3. 10.1371/journal.pone.0174251 PMC5407781 28448582

[90] Dutra MT Reis DBV Martins KG Comparative evaluation of adiposity indices as predictors of hypertension among brazilian adults. Int J Hypertens 2018;2018:1–7. 10.1155/2018/8396570 PMC6009023 29971160

[91] Campo G Punzetti S Malagu M Two-year outcomes after first- or second-generation drug-eluting stent implantation in patients with in-stent restenosis. A PRODIGY trial substudy. Int J Cardiol 2014;173:343–5. 24680246 10.1016/j.ijcard.2014.03.028

